# Assessing metal-induced glycation in French fries

**DOI:** 10.1093/mtomcs/mfae059

**Published:** 2024-12-30

**Authors:** Seth Nobert, Haley Wolgien-Lowe, Tamara Davis, Emma Paterson, Thérèse Wilson-Rawlins, Makan Golizeh

**Affiliations:** Department of Environmental and Physical Sciences, Faculty of Science, Concordia University of Edmonton, Edmonton, Alberta, Canada; Metals in Environment and Health (MEH) Research Cluster, Concordia University of Edmonton, Edmonton, Alberta, Canada; Department of Environmental and Physical Sciences, Faculty of Science, Concordia University of Edmonton, Edmonton, Alberta, Canada; Metals in Environment and Health (MEH) Research Cluster, Concordia University of Edmonton, Edmonton, Alberta, Canada; Department of Environmental and Physical Sciences, Faculty of Science, Concordia University of Edmonton, Edmonton, Alberta, Canada; Metals in Environment and Health (MEH) Research Cluster, Concordia University of Edmonton, Edmonton, Alberta, Canada; Department of Environmental and Physical Sciences, Faculty of Science, Concordia University of Edmonton, Edmonton, Alberta, Canada; Metals in Environment and Health (MEH) Research Cluster, Concordia University of Edmonton, Edmonton, Alberta, Canada; Department of Environmental and Physical Sciences, Faculty of Science, Concordia University of Edmonton, Edmonton, Alberta, Canada; Metals in Environment and Health (MEH) Research Cluster, Concordia University of Edmonton, Edmonton, Alberta, Canada; Department of Environmental and Physical Sciences, Faculty of Science, Concordia University of Edmonton, Edmonton, Alberta, Canada; Metals in Environment and Health (MEH) Research Cluster, Concordia University of Edmonton, Edmonton, Alberta, Canada

**Keywords:** non-enzymatic glycation, chelation, iron, French fries, phytic acid, rice bran

## Abstract

Non-enzymatic glycation is the chemical reaction between the amine group of an amino acid and the carbonyl group of a reducing sugar. The final products of this reaction, advanced glycation end-products (AGEs), are known to play a key role in aging and many chronic diseases. The kinetics of the AGE formation reaction depends on several factors, including pH, temperature, and the presence of prooxidant metals, such as iron and copper. In this study, the effect of iron and copper on the rate and outcome of non-enzymatic glycation was examined in the test tube and a food model, using chromatography and spectrometry methods. Binding efficiencies of several chelating agents to selected metals were also assessed. Phytic acid was the most efficient of the tested chelating agents. The effect of phytic acid on AGE formation in French fries was evaluated. While phytic acid treatment increased the amounts of UV-absorbing compounds in fries, a food ingredient rich in phytic acid showed the opposite effect. This study suggests that prooxidant metals can affect the rate, outcome, and yield of the non-enzymatic glycation reaction and that they do so differently when free or chelated. Moreover, despite being an excellent iron chelator, phytic acid can promote AGE formation in fried food potentially via mechanisms other than metal-induced glycation.

## Graphical Abstract

**Graphical Abstract:**
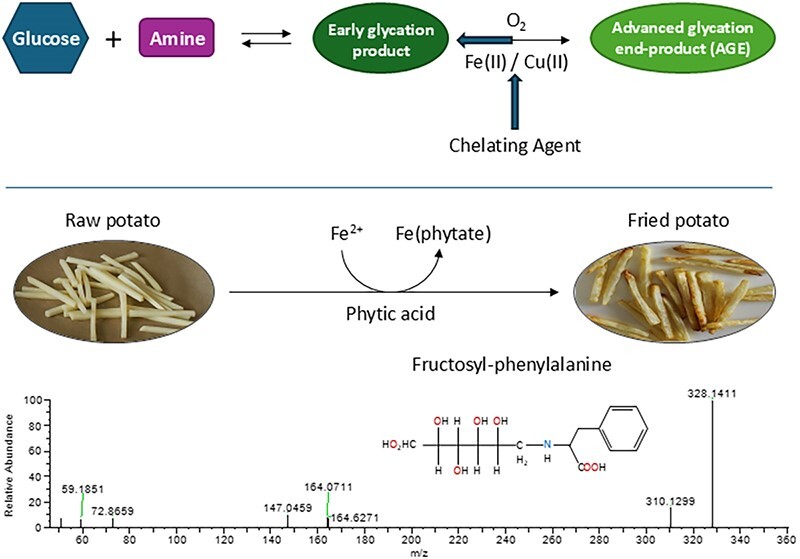

## Introduction

Non-enzymatic glycation is the condensation reaction between the free amine group of a molecule, such as a protein, and the carbonyl group of a reducing sugar, such as fructose or glucose. In contrast to glycosylation, in which a carbohydrate or glycan is enzymatically linked to an N or O atom, glycation occurs when a carbohydrate moiety is attached to an N atom in a non-enzymatic manner. A glycation product can undergo further chemical reactions to produce a myriad of elimination, cyclization, and cross-linking products, collectively known as advanced glycation end-products (AGEs). Several groups of glycation products have been characterized, including early glycation products (e.g. fructosyl amino acids), monolysyl derivatives (e.g. carboxymethyl-lysine, CML), cyclic AGEs (e.g. hydroimidazolones), fluorescent AGEs (e.g. pyrimidine and pentosidine compounds), and cross-linked AGEs (e.g. glucosepane, glyoxal- and methylglyoxal-derived lysine dimers) [1]. AGEs are clinically important as they can bind to reactive sites of other molecules causing covalent modification and cross-linking-induced aggregation, leading to apoptosis, hypersensitivity, and plaque formation [2]. Increased AGE levels have been correlated with various chronic conditions, including cancer, cardiovascular diseases, diabetes, immunological diseases, infections, and neurodegenerative diseases [3].

AGE formation is an oxidative reaction, and increases in the presence of oxidation promoters, such as reactive oxygen species (ROS) [4]. Transition metals, e.g. iron and copper, catalyze ROS production by facilitating redox reactions thus increasing the rate of the AGE formation reaction, which is otherwise a slow reaction under biological conditions [2]. It is for this reason that metal chelators are believed to have a strong inhibitory effect on AGE formation [5]. Chelating agents have been used as detoxification and therapeutic agents for a broad range of medical conditions [6]. Ethylenediaminetetraacetic acid (EDTA) is the most widely used metal chelator, which binds calcium, trace elements, and most transition metals. However, due to its high ligation ability and low selectivity, EDTA treatment can lead to serious side effects via excessive depletion of essential metals [7]. In contrast, natural chelating agents (NCAs), abundant in fruits and vegetables, offer the same chelation ability more selectively [8]. Polyacids, polyphenols, and polythiols, such as phytic acid, curcumin, and phytochelatins, respectively, are among the most studied NCAs.

In foods, non-enzymatic glycation is one of the sources of browning seen by cooking methods, such as frying and baking, and AGEs are formed alongside the compounds that give aroma and colour to the food [9]. The amine groups involved in dietary AGE formation are found in lipids, nucleic acids, and proteins. The quantity of AGEs produced during cooking depends on multiple factors, including cooking method, moisture level, pH, temperature, time, and the fat content of the food [10]. It has been found that increased pH, temperature, and fat content all enhance AGE formation, and that more AGEs are found on the outer surface of foods [11]. Increased moisture content, however, reduces AGE formation [12]. Thus, AGE formation can be reduced by cooking methods that use high moisture, such as boiling, poaching, and stewing. Additionally, acidic pH and the addition of antioxidants can help decrease AGE formation [10]. AGEs are also more readily formed at higher temperatures [13]. The loss of amino acids during AGE formation diminishes the nutritional value of the food, and cross-linking of proteins can lower their digestibility [14].

AGEs are notoriously difficult to analyze as they comprise a wide variety of structures, grouped together only by their mechanism of formation. They can undergo numerous reactions and rearrangements and thus range in size from simple molecules to complex, cross-linked proteins. Moreover, AGEs have limited UV absorbance and are often present in complex sample matrices. Most AGEs are not commercially available to purchase, and synthesis is difficult and gives low yields, limiting the use of standards for identification and/or quantitation [9]. To minimize these complications, CML is often chosen for analysis to represent the overall incidence of AGEs in biological samples [15]. This method is a compromise that can under- or overestimate true AGE content in a sample. The most common analysis methods are enzyme-linked immunosorbent assay (ELISA) and high-performance liquid chromatography (HPLC) with a fluorescence detector (FLD) or coupled with mass spectrometry (MS) or tandem MS (MS/MS) [1]. These methods can be inaccurate or nonspecific, such as with ELISA, expensive and time-consuming, as with HPLC-FLD and HPLC-MS/MS, or limited in the number of detectable analytes, such as with HPLC-FLD, as it requires compounds to be fluorescent. Alternatively, gas chromatography (GC) can be performed on low-molecular-weight AGEs after derivatization to induce volatility. However, unlike HPLC, GC is a destructive method, can be only used for small molecules (usually <400 Da) and is, therefore, not as commonly used for the analysis of AGEs [13].

In this study, we have developed methods for small-scale synthesis and quantitation of metal-induced glycation products. Preliminary tests were made with a simple reaction mixture, containing a reducing sugar (d-glucose) and an amino acid (l-lysine), with and without iron or copper. The effect of various reaction parameters on non-enzymatic glycation was assessed. An HPLC method was developed to quantify metal-induced glycation products in a French fry model cooked with and without phytic acid or its natural source, rice bran, as a dietary NCA. Liquid chromatography (LC)-MS/MS was then used to identify glycation products formed in the simple mixture and the French fries cooked under different conditions. X-ray fluorescence (XRF) spectrometry was also used to assess the chelation efficiency of EDTA and several NCAs with selected transition metals.

## Methods

### Chemicals and reagents

All chemicals and reagents were analytical grade, except HPLC solvents, which were chromatography grade. EDTA, hexane, dl-malic acid, phytic acid, sodium borohydride, sodium citrate tribasic dihydrate, and l-tartaric acid were purchased from Sigma–Aldrich (St. Louis, MI). All other reagents were purchased from Fisher Scientific, (Waltham, MA). Canola oil, russet potato, and table salt were purchased from President's Choice (Brampton, ON). Ultrapure water was obtained from a Millipore (Burlington, MA) Milli-Q IQ 7000 water system. Metal (II) chloride adducts (MCl_2_) were used to produce M^2+^ ions in solution.

### Non-enzymatic glycation in test tubes

**Figure 1.**
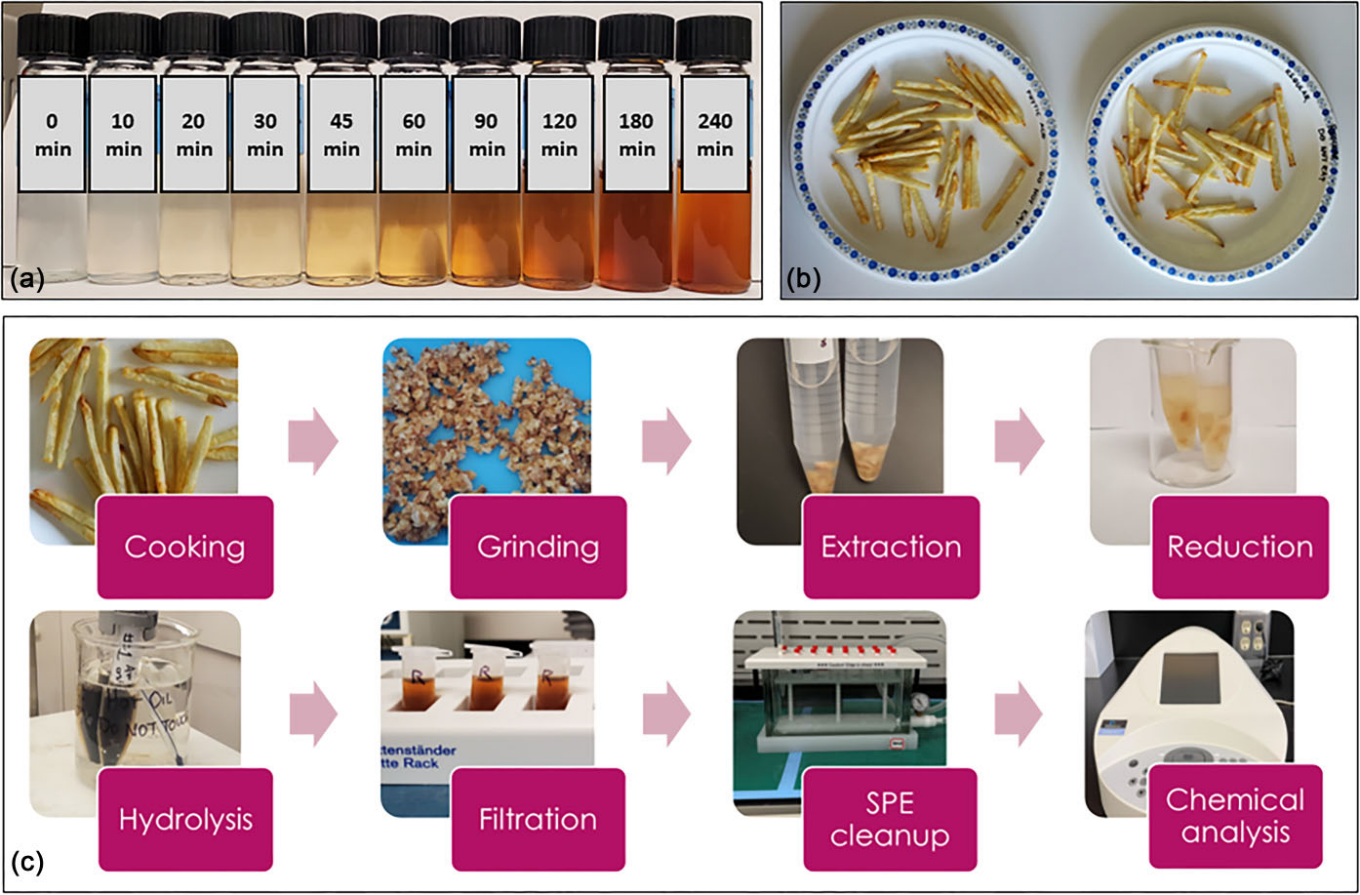
(a) Non-enzymatic glycation in test tubes. D-Glucose and L-lysine were allowed to react in the presence of iron(II) under reflux conditions at 100°C and pH 7.4 for 4 h. Samples were taken for chemical analysis at various time points between 0 and 4 h. (b) Phytic acid-treated (left) and untreated (right) French fries were weighed and transferred to clean paper plates for sensory analysis. (c) The analytical workflow for sample preparation and chemical analysis of French fries.

Three methods were used to synthesize glycation products in test tubes: slow incubation (8 weeks, 37°C), microwave (MW) irradiation (10 min, 420 W), and aqueous-based reflux (4 h, 100°C). d-Glucose (10.0 mM) and l-lysine (10.0 mM) were heated with or without Fe^2+^ or Cu^2+^ (1.00 mM) using one of the three reaction conditions. Reaction time, pH, and MW power output were adjusted to maximize glycation product formation. Reaction progress was monitored by thin-layer chromatography (TLC) using 1% acetic acid in H_2_O and silica gel 60 F_254_ (Millipore) as the mobile and stationary phases, respectively. MW-assisted reactions were performed on 0.6 g of silica gel 60 (0.036–0.071 mm, 215–400 mesh, Alfa Aesar) wetted with ∼1.0 ml H_2_O in 50-ml porcelain crucibles using a Galanz (Foshan, China) countertop MW oven (model: GLCMKA07RDR-07). Reaction temperature was monitored using a Wintact (Shenzhen, China) WT327A infrared laser thermometer. Slow incubation and MW-assisted reactions were performed in 1.5- or 5.0-ml plastic tubes. Reflux reactions were conducted in 250-ml borosilicate round-bottom flasks and sampled at 0, 10, 20, 30, 45, 60, 90, 120, 180, and 240 min (Fig. 1a). To control the effect of heavy metals on non-enzymatic glycation, ultrapure water (18 MΩ.cm at 25°C) was used for all reactions. Each reaction was paired with a control reaction with no metal added. Samples were stored at −20°C prior to analysis.

### Spectrophotometric measurement of glycation products

Absorbance was measured at 295 ± 5 nm in a 0.1-cm quartz cuvette using a PerkinElmer, Inc. (Waltham, MA) Lambda XLS UV-visible spectrophotometer. Samples were diluted if absorbance >1.5. Fluorescence emission was measured at 491 nm using a Wilson Analytical (St. Albert, AB) Open Platform System with a 365-nm LED light source, Ocean Optics Flame miniature spectrometer, and OceanView software (version 1.6.7 Lite). Measurements were performed in triplicate (*n* = 3).

### Chemical derivatization of glycation products

Selected samples were derivatized with *N*-benzoyloxy succinimide (*N*-BOS) using a modification of a published method [16]. Briefly, 250.0 μl of 0.45 μm nylon-filtered sample was incubated (30 min, 25°C) with 1.500 ml of 50 mM borate buffer pH 9.0 in 5% acetonitrile (v/v) and 1.000 ml of 20 mM *N*-BOS in acetonitrile. The derivatized sample was analyzed the same day by HPLC. Samples were stored at 4°C when not in use. Two other amine derivatization methods were tested using *ortho*-phthalaldehyde or phthalic anhydride; however, the *N*-BOS method was selected due to the higher reproducibility.

### Preparation of metal chelation samples

Stock solutions of 2000 ppm were prepared for each metal (Cd, Co, Cu, Fe, Ni, Pb, and Zn), using metal (II) chloride adducts, as well as for each chelating agent (citric acid, EDTA, malic acid, oxalic acid, phytic acid, and tartaric acid) in ultrapure water. Then, 500 μl metal (1000 ppm) and 500 μl chelating agent (1000 ppm) were incubated (60 min, 80°C) in a 1.5-ml plastic tube and allowed to cool. Samples were then subjected to one of the two methods to separate complexed and free metals. For clear solutions, cation exchange solid-phase extraction (SPE) was employed using an Oasis mixed-mode cation exchange (MCX) SPE cartridge (Waters Corporation, Milford, MA) with 1.0 M HCl elution and a Supelco (Bellefonte, PA) SPE vacuum system. The unbound fraction containing complexed metal and the eluate containing free metal ions were collected in 1.5-ml plastic tubes. For non-clear solutions and suspensions, acid digestion was used to quantify the complexed metal. Briefly, the sample was centrifuged (10 min, 15 000×*g*). The supernatant (free metal ions) was then transferred into a clean 1.5-ml plastic tube. The precipitate (complexed metal) was digested in 6.0 M HCl at 80°C until a clear solution was obtained.

### XRF analysis of metals in aqueous solutions

Approximately 1.0 ml of a liquid sample was transferred to a plastic weighing dish, and directly analyzed by a Bruker (Billerica, MA) CTX-500 Graphene benchtop XRF spectrometer. The spectrometer was internally calibrated for liquid samples using 5 calibration standards made from each metal of interest at 100, 250, 500, 750, and 1500 ppm. The slope, offset, and *R*^2^ value were calculated for each calibration. XRF measurements were performed in triplicate (*n* = 3).

### Determination of effective formation constants

The uncorrected effective formation constant (*K*_f_*′*) for the M^2+^ + X*^n^*^−^⇌ MX*^n^*^−2^ reaction was calculated based on XRF concentrations of the complexed and free metal using the following equation:


\begin{eqnarray*}
{K^{\prime}}_{\mathrm{f}}\, &=& \,\frac{{\left[ {{\mathrm{M}}{{\mathrm{X}}}^{n - 2}} \right]}}{{\left[ {{{\mathrm{M}}}^{2 + }} \right]{{\mathrm{C}}}_{{{\mathrm{X}}}^{n - }}}}\, \nonumber\\
&=& \,\frac{{{\mathrm{molarity}}\,{\mathrm{of}}\,{\mathrm{complexed}}\,{\mathrm{metal}}}}{{{\mathrm{molarity}}\,{\mathrm{of}}\,{\mathrm{free}}\,{\mathrm{metal}} \times {\mathrm{formality}}\,{\mathrm{of}}\,{\mathrm{chelating}}\,{\mathrm{agent}}}},
\end{eqnarray*}

where M^2+^, X*^n^*^−^, and MX*^n^*^−2^ represent the metal, the chelating agent, and the metal-chelating agent complex, respectively. *K*_f_*′* values were measured at pH∼5.5 and were not corrected for ambient pressure or temperature. *K*_f_′ values were determined for chelating agents added to individual metals but not multiple metals in solution, e.g. iron and copper.

### Preparation of French fry samples

Russet potatoes were chosen for this experiment, sourced from a local supermarket (Save-on-Foods). The preparation step prior to cooking involved rinsing the potatoes under cold tap water, peeling, washing, and then drying using a particle-free towel. Potatoes were then cut into equal-sized portions using a fry cutter and divided further into four. Any potato sample that was not equivalent was then discarded. Fries were then weighed out to 100.00 g, salted (1.00 g) and then either treated with phytic acid (0.66 g), rice bran (3.33 g), or left as control. Samples were cooked (5 min, 375°F), flipped, and then cooked again (5 min, 375°F) in an air fryer (Instant Vortex 6QT, Greencastle, PA). The fries were then collected, and 50.00 g of the sample was blended in a food processor, and a sample of 0.200 g was taken for chemical analysis. The remainder of the unprocessed sample was transferred into clean paper plates for sensory analysis (Fig. 1b).

### Chemical analysis of potato extracts

French fry and raw potato samples were prepared for chemical analysis using a published method [17]. Briefly, the sample was extracted with hexane (3 × 10 ml), centrifuged (10 min, 3170×*g*), reduced with NaBH_4_ (1.00 M in 0.10 M NaOH, overnight), and digested with 2.5 ml 12 M HCl (24 h, 110°C). The digest was filtered and divided into three 2.0-ml aliquots. The aliquots were dried under nitrogen, redissolved in 0.10 M HCl, centrifuged (10 min, 10 000×*g*), and subjected to MCX SPE. Free and complexed Fe^2+^ were measured using XRF and the Fe K_α_ emission line. The SPE eluate was then analyzed by UV-visible spectrophotometry, dried under nitrogen, and redissolved in 0.3% acetic acid in H_2_O for HPLC and MS analyses. Caffeine (in 50% acetonitrile) was added as an internal standard (IS) to a final concentration of 100 ppm. HPLC, UV-visible, and XRF analyses were performed in triplicate (*n* = 3). Figure 1c depicts the sample preparation and chemical analysis workflow.

### HPLC analysis

Sample analysis was performed on an Agilent (Santa Clara, CA) 1260 HPLC system in reverse-phase mode with a diode-array detector (DAD). Briefly, 10.0 μl of the sample was injected on to an Agilent Poroshell 120 EC-C18 column (4.6 × 100 mm, 2.7 μm) at 50°C with a flow rate of 1.000 ml/min. Mobile phases were H_2_O (A) and acetonitrile (B), both with 0.3% glacial acetic acid (v/v). The gradient program increased B from 0% to 70% in 14 min, held B at 70% for 3 min, reduced B to 0% in 0.5 min, and held B at 0% for 10.5 min to re-equilibrate the column for a total run time of 28 min. The response was monitored at 230, 270, and 295 nm.

### MS analysis of incubation, MW, and reflux samples

LC-MS and LC-MS/MS analyses were performed at the University of Alberta Mass Spectrometry Facility. Incubation, MW irradiation, and reflux samples were analyzed by an Agilent 1200 SL HPLC system equipped with an Agilent Poroshell 120 EC-C18 column (4.6 × 50 mm, 2.7 μm) at 50°C with 1.000 ml/min flow rate (injection volume 1.5 μl). The mobile phases were H_2_O (A) and acetonitrile (B), both with 0.1% formic acid (v/v). The gradient program started at 1% B and increased linearly to 95% B over 14 min, followed by a 3-min hold, then reduced to 1% B in 0.5 min. The UV signal was acquired at 230, 270, and 295 nm. The mass spectra were obtained using an Agilent 6220 Accurate-Mass time-of-flight LC-MS system in positive and negative ionization modes with a dual sprayer electrospray ionization (ESI) source, with the second sprayer delivering a reference solution. The HPLC eluent was split ∼50:50 before entering the sprayer. The drying gas was delivered at 10 l/min at 325°C, the nebulizer was 30 psi, mass range of 100–1100 Da, acquisition rate of ∼1.03 spectra/s, fragmentor 130 V for positive mode and 125 V for negative mode. The skimmer was set at 65 V, capillary at 3200 V, and the instrument state was 4 GHz high resolution. Mass correction was performed for every spectrum using the reference peaks (*m*/*z* 121.0509 and 922.0098 in positive mode; 112.9856 and 1033.9881 in negative mode). LC-MS data were acquired with the Agilent MassHunter software.

### MS analysis of potato extracts

Potato extracts were subjected to LC-MS/MS analysis using a Vanquish UHPLC System (Thermo Fisher Scientific, Germering, Germany) coupled to an Orbitrap Exploris 240 mass spectrometer (Thermo Fisher Scientific). HPLC analysis was performed as described above (injection volume 2.0 μl). MS conditions were as follows: sheath gas 60, auxiliary gas 15, sweep gas 2, ion transfer tube at 350°C, vaporizer at 350°C, spray voltage 3500 V (positive mode), and 2500 V (negative mode), and RF lens was at 70%. MS scan resolution was set to 90k with a scan range from 100 to 1100 Da. MS/MS was run by selecting the five most intense peaks with monoisotopic peak detection set to small molecules, dynamic exclusion of peaks after two scans if within 3 s, exclusion duration 5 s with a tolerance of 5 ppm. Isolation window was set to 1.8 Da at a scan resolution of 15k with HCD normalized collision energy of 15%, 25%, and 60%. LC-MS/MS data acquisition and preliminary data analysis were performed using the “unknown screening” feature of the TraceFinder 5.1 (build 203) software (Thermo Fisher Scientific).

### Data analysis

XRF peak integration was performed in the Bruker Instrument Tools (Bruker Nano GmbH, Berlin, Germany) data analysis software (version 1.9.0.146). HPLC peak integration was carried out by the Agilent ChemStation OpenLab CDS software (version 2.14.29). HPLC quantitation was conducted based on the IS peak area (5.3 min @ 295 nm DAD). LC-MS data analysis was performed using the Agilent MassHunter Qualitative Analysis software. Upon determination of molecular formulae, the resulting formulae were searched in ChemSpider (https://www.chemspider.com/) for probable structures. Selection was based on the requirement of an NH_2_ group in the structure as well as lack of a benzene ring. Further LC-MS/MS data analysis was performed using the FreeStyle 1.8 SP2 QF1 (version 1.8.65.0) data visualization software (Thermo Fisher Scientific). MS and MS/MS spectral library searches were done within the HMDB (https://www.hmdb.ca/) public database [18] with parent ion mass and mass/charge tolerances set to 5 ppm and 0.5 Da, respectively. Only singly charged molecular ions were included in the search. LC-MS quantitation was based on the IS peak area (3.9 min; *m*/*z* 195.08763, in positive, or *m*/*z* 193.07307, in negative ionization mode). Linear regression and statistical testing, including Pearson's correlation, two-way ANOVA (analysis of variance; *α* = 0.05), and Student's *t* tests were performed in Microsoft Excel. Grubb's test (*α* = 0.05) was used for outlier detection.

### Sensory analysis of French fries

Rice bran-treated and untreated French fry samples were tested by six volunteers (two females, four males; 21–60 years old) within 20 min of cooking. The volunteers were requested to describe their perception of the appearance, flavour, taste, texture, and smell of the sample, and their comments were recorded. Phytic acid-treated fries were not tested.

## Results

In this study, experiments were conducted to study the chemistry of metal-induced glycation. Methods were developed for laboratory synthesis and chemical analysis of glycation products in the presence of iron or copper. Products formed with and without iron or copper were identified and quantified under various experimental conditions using chromatographic and spectrometric methods. Furthermore, the effect of metal chelators on selected transition metals was assessed. Phytic acid was the most versatile chelator under the studied conditions. A French fry model was later developed to test the effect of phytic acid on metal-induced glycation at elevated temperatures. The following sections summarize the results of each experiment.

### Synthesis of non-enzymatic glycation products in test tubes

**Figure 2.**
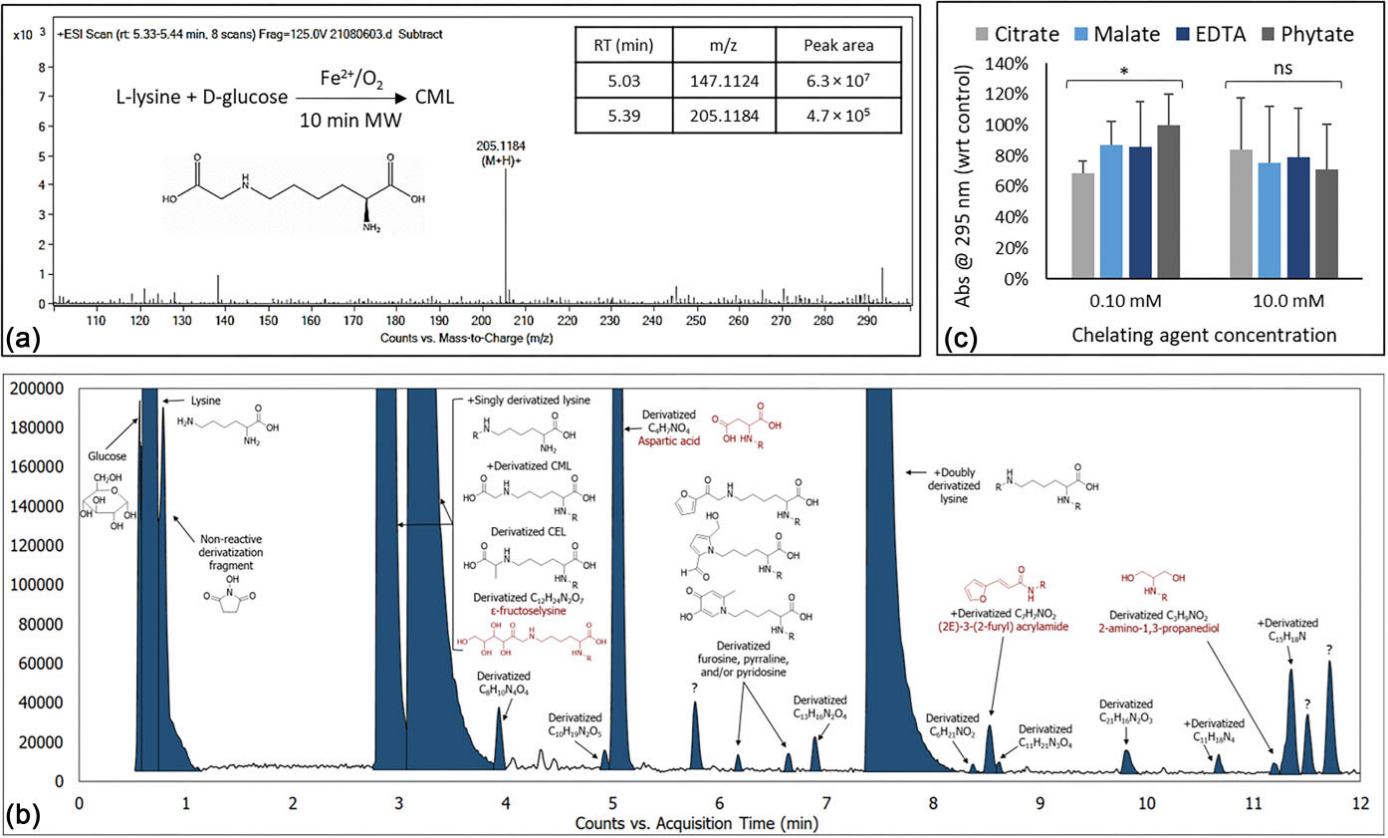
(a) Positive-mode ESI mass spectrum and LC-MS peak areas from the most abundant constituents in the non-enzymatic glycation *in vitro* reaction mixture. (b) Base peak chromatogram of the reaction mixture derivatized with N-benzoyloxy succinimide in negative-mode ESI LC-MS. (c) Effect of selected chelating agents on non-enzymatic glycation product formation in the presence of Fe^2+^ (10 min MW irradiation at 420 W energy output). Absorbance of the reaction mixture is presented with respect to the control/no metal sample. Error bars represent standard deviation. Asterisk and ns represent *P* < 0.05 and *P* > 0.05 (ANOVA), respectively. CML, carboxymethyl- lysine; CEL, carboxyethyl-lysine; R, derivatization fragment; RT, retention time; +, also found in positive-mode ESI-LC-MS; ?, unidentified compound. Compounds 2-amino-1,3-propanediol, aspartic acid, ε-fructoselysine, and (*2E*)-3-(2-furyl) acrylamide are tentative MS library matches only and have not been confirmed.

Several reaction conditions were tested to maximize the yield of the glycation reaction in test tubes. d-Glucose and l-lysine were reacted at 1:1 or 2:1 molar ratio with and without Fe^2+^ or Cu^2+^ at a 1:10 molar ratio with respect to lysine. Various pH values, temperatures, and reaction conditions were also tested, e.g. slow incubation versus MW-assisted synthesis versus aqueous-based reflux. The reaction progress was monitored by TLC and UV-visible spectrophotometry. The wavelength of maximum absorbance (λ_max_) was 295 nm. This wavelength was, therefore, used as a surrogate for the total quantity of glycation products in the solution. Neither starting material absorbs at this wavelength. In summary, the glucose/lysine molar ratio did not significantly impact total glycation product concentration. Neutral pH (7.4) was a more favorable reaction condition than slightly basic pH (8.5). No measurable glycation was detected at pH 4.5. Iron yielded more glycation products compared to copper or controls (Abs_295_ 1.75 ± 0.02 for iron vs. 1.65 ± 0.02 for copper vs. 1.25 ± 0.02 for control at pH 7.4). Four weeks of incubation at 37°C, 10 min MW irradiation at 420 W energy output, and 4 h of reflux under aqueous conditions (100°C) resulted in comparable total glycation product concentrations. LC-MS confirmed the major constituent of the product mixture to be CML. Figure 2a exhibits the positive-mode ESI-LC-MS spectrum from CML (*m*/*z* 205.1184) via MW-assisted synthesis. LC-MS peak areas from CML and unreacted lysine (*m*/*z* 147.1124) are also listed. Other AGEs and oxidation products tentatively identified in the product mixture, using the *N*-BOS derivatization method and LC-MS, were carboxyethyl-lysine (CEL), fructosyl-lysine, (2*E*)-3-(2-furyl)-acrylamide, 2-amino-1,3-propanediol, furosine, pyrraline and/or pyridosine, as well as aspartic acid (Fig. 2b).

### Effect of metal chelation on non-enzymatic glycation in test tubes

Total glycation product concentration (based on Abs _295_) of iron- or copper-treated samples radiated (MW) with 0.10 or 10.0 mM of citric acid, malic acid, phytic acid, or EDTA were compared with that of untreated (no added iron or copper) samples. Samples treated with chelating agents had lower absorbance than the untreated samples. Citric acid and phytic acid yielded the lowest absorbances at 0.10 mM and 10.0 mM chelating agent concentrations, respectively. Samples treated with 0.10 mM citric acid, 10.0 mM malic acid, or 10.0 mM phytic acid had lower absorbance at 295 nm than those treated with EDTA, as shown in Fig. 2c. To stay within physiologically feasible ranges, higher chelating agent concentrations were not tested. CML remained the major constituent of the reaction mixture under chelating agent treatment conditions.

### Chelation efficiency of NCAs with heavy metals

Effective formation constants were determined for five NCAs, as well as EDTA, as a comparator, with Cd^2+^, Co^2+^, Cu^2+^, Fe^2+^, Ni^2+^, Pb^2+^, and Zn^2+^ using XRF. The NCAs included in this study were citric acid, malic acid, oxalic acid, phytic acid, and tartaric acid. Phytic acid had higher *K*_f_*′* values than EDTA with all metals, except Co^2+^, ranging from 3.02 × 10^2^ to 1.29 × 10^5^. Citric acid and oxalic acid demonstrated lower chelating abilities than EDTA. However, they had higher *K*_f_*′* values than phytic acid for Co^2+^ (4.51 × 10^2^ and 1.03 × 10^3^, respectively, compared to 3.02 × 10^2^ for phytic acid). Citric acid was also a better chelating agent than phytic acid for Ni^2+^ (*K*_f_′ = 4.01 × 10^2^ vs. 3.59 × 10^2^ for phytic acid). Phytic acid was the only NCA showing a chelating ability for Cu^2+^ (*K*_f_′ = 2.12 × 10^4^) and also with the highest affinity toward Zn^2+^ (*K*_f_′ = 1.22 × 10^4^). Table 1 lists *K*_f_*′* values for each metal chelation reaction.

**Table 1.** Effective formation constant (*K*_f_*′*) values calculated for selected chelating agents and heavy metals using X-ray fluorescence (XRF) spectrometry. *K*_f_*′* values were not corrected for ambient pressure or temperature. < LOD: below the limit of detection. Least-squares regression R^2^ value for XRF internal calibration of each metal is included.

**Chelating agent**	**Cd^2+^**	**Co^2+^**	**Cu^2+^**	**Fe^2+^**	**Ni^2+^**	**Pb^2+^**	**Zn^2+^**
(least-squares R^2^)	(0.97)	(0.99)	(0.99)	(0.99)	(0.99)	(0.96)	(0.99)
Citric acid	<LOD	4.51 × 10^2^	<LOD	6.92 × 10^3^	4.01 × 10^2^	1.48 × 10^4^	2.70 × 10^2^
EDTA	<LOD	2.97 × 10^3^	1.07 × 10^4^	7.72 × 10^4^	<LOD	1.66 × 10^4^	7.90 × 10^3^
Malic acid	<LOD	8.02 × 10^1^	<LOD	<LOD	3.33 × 10^2^	<LOD	<LOD
Oxalic acid	<LOD	1.03 × 10^3^	<LOD	2.63 × 10^4^	<LOD	1.56 × 10^4^	2.49 × 10^2^
Phytic acid	6.65 × 10^2^	3.02 × 10^2^	2.12 × 10^4^	1.29 × 10^5^	3.59 × 10^2^	3.46 × 10^4^	1.22 × 10^4^
Tartaric acid	<LOD	<LOD	<LOD	<LOD	1.48 × 10^5^	<LOD	<LOD

### Effect of phytic acid on non-enzymatic glycation in French fries

**Figure 3.**
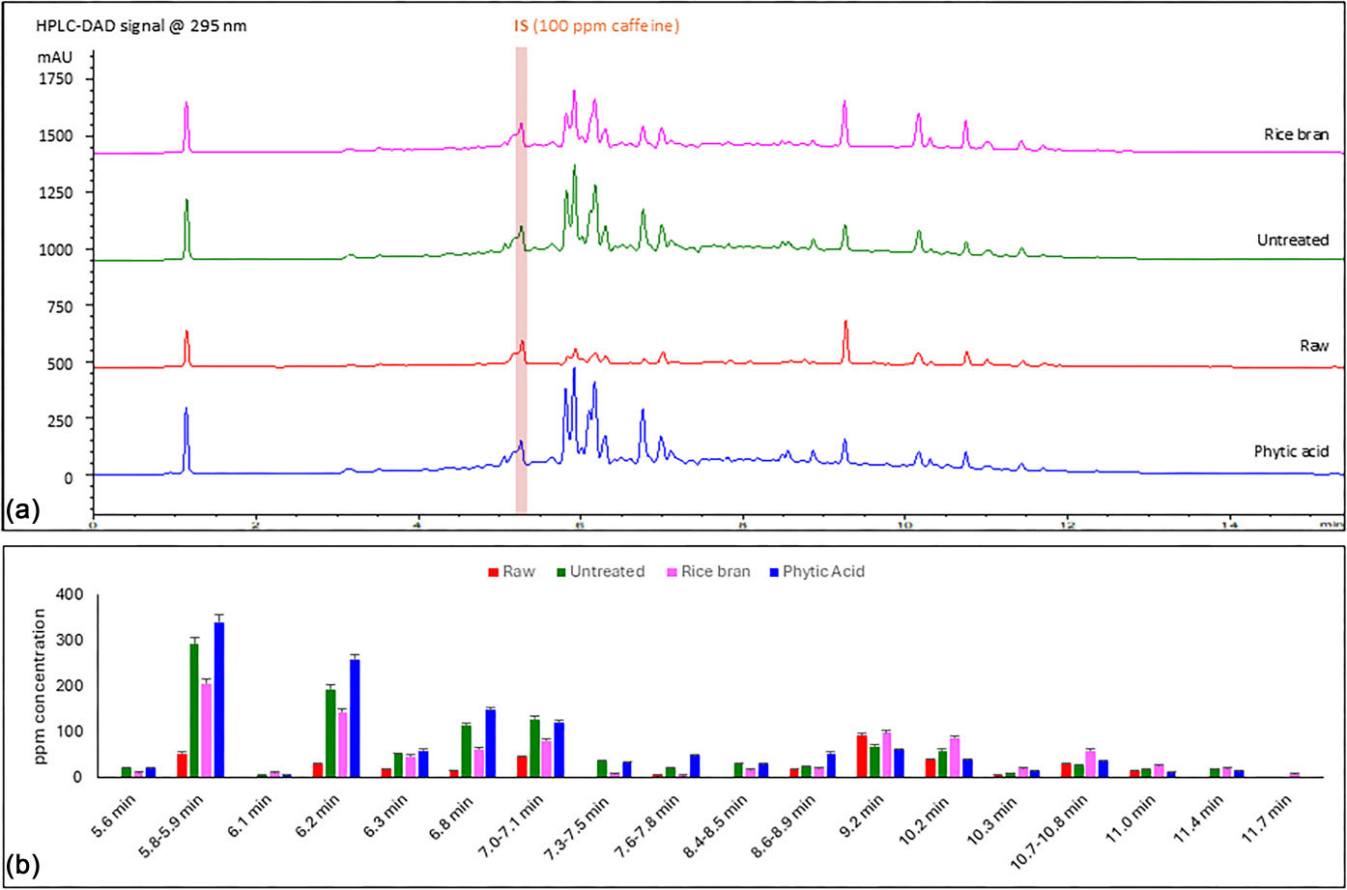
(a) HPLC-DAD chromatograms from raw, air-fried untreated, air-fried treated with rice bran, and air-fried treated with phytic acid potato samples recorded at 295 nm. (b) Retention time distribution and total ppm concentration of UV-absorbing compounds in the four potato samples. IS peak (at 5.2 min) and poorly retained substances (retention time < 5.0 min) were omitted. Error bar represents standard deviation.

HPLC-DAD analysis of potato extracts from the following conditions were compared: raw potato, air-fried potato, air-fried potato treated with phytic acid (6.6 mg/g potato), and air-fried potato treated with rice bran (33.3 mg/g potato), a rich natural source of phytic acid. Figure 3a exhibits HPLC-DAD chromatograms (at 295 nm) from each sample. Most detected compounds eluted from the C18 reverse-phase HPLC column between 5.8 and 7.1 min. Compounds eluting at 5.8–5.9 min had the highest absorbance at 295 nm in all samples (Fig. 3a and b). The phytic acid-treated sample yielded the highest total quantity of 295 nm-absorbing species (1500 ± 100 ppm) compared to the raw potato sample (462 ± 6 ppm) followed by the untreated sample (1400 ± 100 ppm). The rice bran-treated sample contained the lowest total quantity of 295 nm-absorbing compounds in all air-fried samples (1086 ± 3 ppm; ANOVA *P* = 3.4 × 10^−7^; Table 2). Air frying altered the retention time distribution of 295 nm-absorbing compounds in the potato extract (i.e. raw vs. air fried), particularly at 5.5–7.5 min. However, rice bran or phytic acid treatment did not show any observable effect on the peaks in this region in the air-fried samples (Fig. 3b). Air frying increased the iron content of the potato sample from 50 ± 80 to 190 ± 70 ppm. However, phytic acid-treated and rice bran-treated samples had lower iron contents than the untreated air-fried sample (160 ± 80 and 90 ± 80 ppm, respectively; ANOVA *P* = 9.7 × 10^−7^; Table 2).

**Table 2.** Total 295 nm-absorbing species (HPLC-DAD) and iron (XRF) in prepared extracts from raw, air-fried, air-fried treated with phytic acid, and air-fried treated with rice bran potato samples. HPLC quantitation was performed using caffeine (100 ppm) as an internal standard. The analysis was performed in triplicate (n = 3). Values are mean ± standard deviation

**Potato**	**ppm**	**ppm**
**sample**	**295 nm-absorbing**	**Fe**
**(100.00 g)**	**species (HPLC-DAD)**	**(XRF)**
Raw	462 (±6)	50 (±80)
Fried	1400 (±100)	190 (±70)
Fried with phytic acid	1500 (±100)	160 (±80)
Fried with rice bran	1086 (±3)	90 (±80)

### Sensory analysis of rice bran-treated French fries

All volunteers stated that they appreciated the appearance, flavour, taste, texture, and smell of untreated (regular) French fries. Their assessment of the rice bran-treated fries was that they had a different flavour and taste compared to untreated fries. Descriptive comments included rice bran-treated fries having a sweet flavour with late salt, as well as a metallic or mineral aftertaste. Volunteers did not perceive any difference in appearance, texture, or smell between the treated and untreated samples.

### Molecular profile of phytic acid-treated French fries

**Figure 4.**
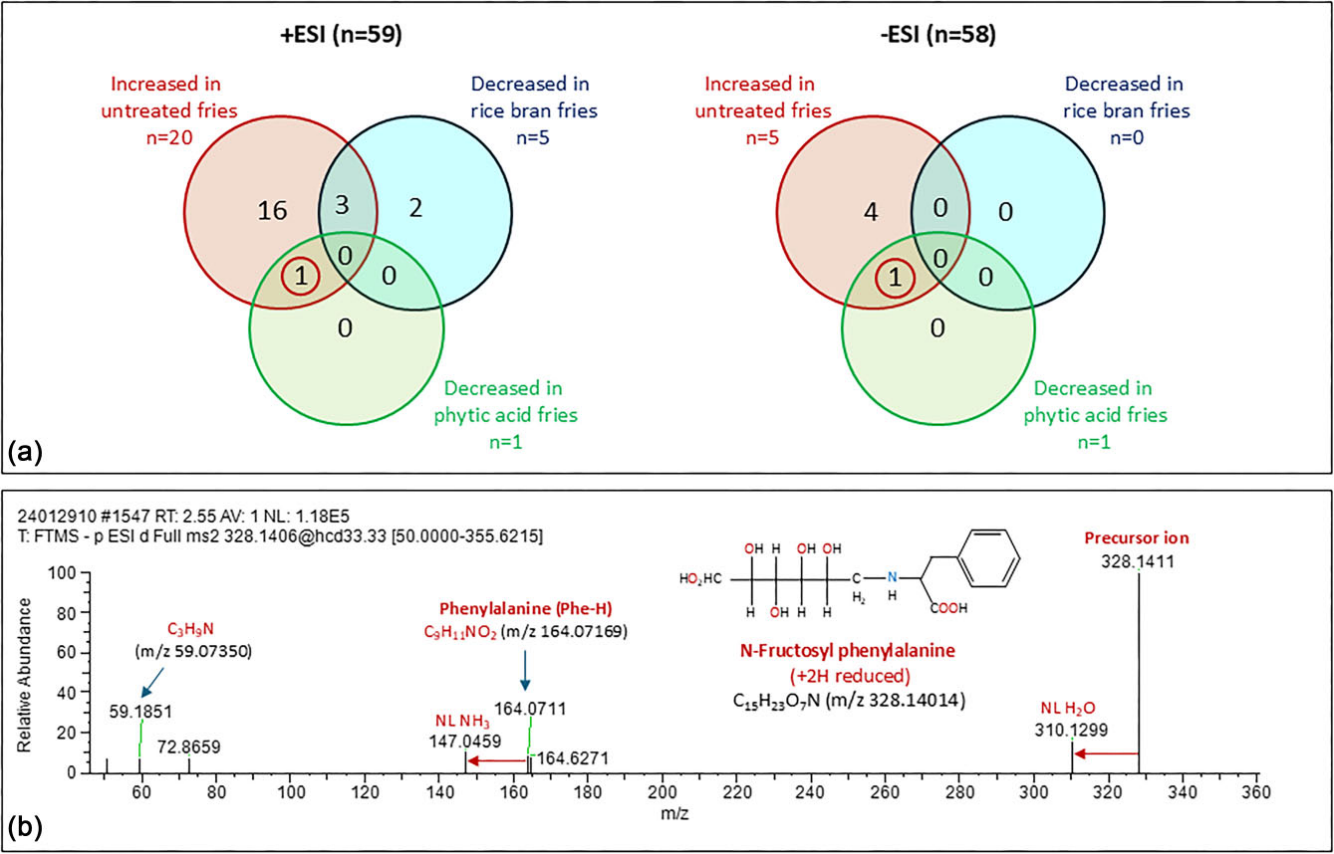
(a) Venn diagrams demonstrating the number of LC-MS features altered in untreated, phytic acid-treated, and rice bran-treated French fries (compared to raw potato) in positive (left) and negative (right) ESI modes. Out of the 117 detected compounds, 25 increased >100% in untreated French fries compared to raw potato, 5 decreased to raw potato levels in rice bran- treated, and 1 decreased in phytic acid-treated fries. The latter compound (circled) was identified, in both positive-mode and negative-mode ESI, as fructosyl-phenylalanine, an early glycation product of phenylalanine. (b) Negative-mode ESI high-resolution MS/MS spectrum and tentative molecular structure of the compound decreased in phytic acid-treated French fries. NL, neutral loss.

A total of 498 LC-MS peaks were detected in samples of raw potato, untreated, phytic acid-treated, and rice bran-treated French fries (255/243, +ESI/−ESI). The list was trimmed down to 117 compounds, including the IS, after duplicate removal (59/58; +ESI/−ESI). Out of these, 25 compounds increased in untreated fries (20/5; +ESI/−ESI), 5 increased in phytic acid-treated fries (4/1; +ESI/−ESI), 11 increased in rice bran-treated fries (4/8; +ESI/−ESI), and 4 increased with both treatments (3/1; +ESI/−ESI). Moreover, 1 compound out of 117 decreased in phytic acid-treated fries (detected in both +ESI and −ESI), and 5 decreased in rice bran-treated fries (5/0; +ESI/−ESI; Fig. 4a). The words increase or decrease refer to a fold-change ≥ 2, i.e. LC-MS peak intensity change ≥ 100% with respect to the raw potato sample. +ESI and −ESI are short for positive-mode and negative-mode ESI MS. Many compounds had at least one MS spectral library match within 5 ppm mass accuracy. However, only three compounds resulted in an MS/MS spectral library match (5 ppm MS and 0.5 Da MS/MS mass accuracies). These compounds were fructosyl-phenylalanine (increased in untreated and rice bran-treated but decreased in phytic acid-treated fries; Fig. 4b), bitalin A (increased in rice bran-treated fries), and anofinic acid (decreased in all types of fries but less in rice bran-treated fries). The LC-(high-resolution)–MS/MS data of these compounds are included in Figs S1–S3.

## Discussion

Metal-induced oxidation plays a key role in human health [19]. It is also one of the mechanistic causes of AGE formation [20–22]. Another source of AGEs is nucleophilic addition of free amines to the ROS [2], whose formation is also catalyzed by prooxidant metals [23]. Understanding the link between metal-induced oxidation and AGE formation has, therefore, been a growing interest over the past years [24]. In this study, we focused on two of the most endogenously abundant prooxidant metals in humans, iron and copper, and their potential impact on non-enzymatic glycation in a test-tube model. Iron is a stronger oxidant than copper (cf. $E_{{\mathrm{F}}{{\mathrm{e}}}^{2 + }|{\mathrm{Fe}}}^{\mathrm{o}}$ = −0.44 V vs. $E_{{\mathrm{C}}{{\mathrm{u}}}^{2 + }|{\mathrm{Cu}}}^o$ = 0.34 V). This is consistent with our results, as iron yielded more glycation products than copper with d-glucose and l-lysine at pH 7.4 (Abs_295_ 1.75 ± 0.02 for Fe vs. 1.65 ± 0.02 for Cu). Iron is also more abundant in humans than copper (cf. 11–32 μM Fe_total_ vs. 10–22 μM Cu_total_; Medscape). Moreover, iron chelators have shown promise as therapeutic agents for many diseases, highlighting the clinical importance of iron [25]. For these reasons, we selected iron as our primary target in the study of metal-induced glycation. Since potato is a rich source of amino acids, proteins, reducing sugars, and iron, a French-fry model was deemed suitable for this study. French fries are also rich in AGEs due to their relatively high cooking temperatures [26].

AGE formation is efficient and rapid at over 120°C but much slower *in vivo* [4]. Spectrophotometric measurements showed that 4 weeks of incubation at 37°C produce roughly the same amounts of AGEs as 4 h of aqueous reflux (100°C) or 10 min of MW irradiation (∼150°C). Our typical yields for all tested methods (incubation, MW, and reflux) were <1% (Fig. 2a). This amount is approximately equivalent to 20.4 ppm CML, which is about 1/4 of the lower limit of normal for plasma CML in healthy humans (81–298 ppm) [27]. Other glycation products, such as CEL, fructosyl-lysine, and cyclic AGEs were identified in the synthetic samples at much smaller concentrations than CML (Fig. 2b). We were not able to identify CML in the French fry samples. However, an early glycation product, fructosyl-phenylalanine, was found in all potato samples (2.43:39.0:40.4:14.2 ppm; raw potato/untreated/rice bran-treated/phytic acid-treated fries; Fig. 4b and Fig. S1). Potato is among the top 10 richest sources of phenylalanine, with a mean concentration of 7.1 ppm [28]. Therefore, it is not surprising to find this glycated amino acid in French fries. Fructosyl-phenylalanine has been identified in other types of food, such as honey [29], pepper [30], and tomato [31]. It has also been listed as a biomarker for childhood short stature [32] and SARS-CoV-2 infection [33].

Myo-inositol-1,2,3,4,5,6-hexakisphosphoric acid (IP6), commonly known as phytic acid, is the primary source of inositol and phosphate in grains and plant seeds during the maturation period [34]. It is also a powerful NCA with a strong affinity for calcium, iron, magnesium, and zinc cations [35]. Phytic acid is, therefore, sometimes frowned upon for its potential implication in micronutrient depletion [36]. However, recent studies on the interaction between phytic acid and human diet and health suggest that it can be considered safe and may be even beneficial to human health [37]. Metal phytates are water insoluble and cannot participate in aqueous redox reactions. Our measurements showed that phytic acid had the highest effective formation constant with iron among the studied chelators (Table 1) and was able to decrease glycation product formation at a relatively high chelator concentration (Fig. 2c). The lower abundance of fructosyl-phenylalanine in phytic acid-treated fries could be the result of decreased glycation levels due to impaired iron-induced oxidation in the presence of phytic acid because of its high iron chelation ability. However, rice bran-treated fries did not show the same effect.

Rice bran is one of the richest natural sources of phytic acid with up to 8.7% mean phytate content [38]. Recent studies suggest that rice bran and its by-products may have favorable health effects on metabolic conditions and oxidative stress. However, it is not clear whether these effects are related to phytic acid, as rice bran also contains phenolic compounds, such as ferulic acid esters and α-tocopherol (vitamin E) [39]. Our results showed that compared to untreated (regular) French fries, rice bran-treated fries had 22.4(±7.3)% less (ANOVA *P* = 3.4 × 10^−^^7^) compounds absorbing at 295 nm, which is the λ_max_ of d-glucose/l-lysine reaction products based on our test-tube experiments (see “Results” section). We have used total absorption at 295 nm (Abs_295_) as a surrogate to assess total AGE levels in the studied samples. Rice bran-treated fries also had 52.6(±59.2)% less (ANOVA *P* = 9.7 × 10^−^^7^) iron than the untreated fry samples. Phytic acid treatment also decreased the iron content of French fries but not to the same extent (see Table 2). It is possible that the amount of rice bran used contained more phytic acid than anticipated based on the literature. Other chelating agents in rice bran could also form complexes with iron, further disfavoring iron-induced glycation in French fries. We were unable to study the effect of copper on glycation in French fries as, unlike iron, copper is not an abundant metal in potato.

It was surprising to see that phytic acid treatment increased glycation products in French fries by 7.1(±10.1)% (based on Abs _295_) despite decreasing iron by 15.8(±56.2)% compared to the untreated fries. Since phytic acid treatment resulted in lower amounts of the early glycation product fructosyl-phenylalanine (61.7% less than untreated fries), it is more likely that phytic acid could produce other 295-nm absorbing compounds than glycation products e.g. autohydrolysis products [40]. Overall, a very strong positive correlation was found between total glycation product concentration (based on Abs _295_) and total iron in French fries (Pearson's *r* = 0.85), suggesting that iron had a great impact on non-enzymatic glycation.

The addition of rice bran to French fries was found to have tentatively altered the concentration of two other compounds in the fries. Bitalin A had a 27.6-fold increase in rice bran-treated fries and was not detected in phytic acid-treated fires or the raw potato extract (Fig. S2). Bitalin A is a coumaran derivative rarely found in plant species. Little is known about its biological properties except its potential anti-arthritic activity [41]. Bitalin A has not previously been detected in potato or rice bran. Another compound, anofinic acid, had remarkably lower amounts in all French fry samples potentially due to heat-induced degradation; however, it was more abundant in rice bran-treated fries (1.4-fold vs. untreated and 1.5-fold vs. phytic acid-treated samples; Fig. S3). Anofinic acid is a benzopyran derivative and is an extensively studied food-associated compound. It is often derived from endophytic fungi and has been shown to elicit antimicrobial, anticancer, and antibiofilm properties [42]. Fungal endophytes colonize various plants, including potatoes, and develop a balanced, mutually beneficial interaction with the host plant [43]. The antioxidant compounds in rice bran may decrease thermal decomposition of anofinic acid in potato during the frying process, which is potentially why rice bran-treated fries contained more anofinic acid than the other types of fries.

Sensory analysis showed that adding rice bran to potato significantly changes the flavour and taste of the resulting French fries. Testers had mixed feelings about the new recipe and were not fully dissatisfied with it. Recommending rice bran treatment to make a less unhealthy version of French fries is out of the scope of this article and requires a more thorough investigation of the potential impact of this treatment on the molecular composition of fried potato.

The analytical methods developed in this study could be used to assess glycation products in other biological mixtures, such as biofluids, cells, and tissues. Several HPLC elution methods using CN normal-phase, C8 reverse-phase, C18 reverse-phase, and hydrophilic interaction liquid chromatography columns were tested. *N*-BOS derivatization coupled with C18 reverse-phase HPLC provided the most satisfactory elution profile for the test tube mixtures. However, it led to an overly complicated HPLC chromatogram when used for the French fry samples. UV-visible spectrophotometry showed promise as a rapid, convenient method for total AGE quantitation. However, the *λ*_max_ = 295 nm obtained for the products of the glycation reaction is associated with band 2 of the conjugated amine (−C=C−N̈H−) system, which is present in all AGEs, although it is not specific to these compounds. It would be a suitable wavelength for quantitative comparison of glycation products in a mixture only if the analytes are in comparable matrices. Fluorescence measurements at *λ*_excitation_ = 365 nm/*λ*_emission_ = 491 nm were more sensitive and, since they are measured at two wavelengths, would be more specific to the compounds of interest. The SPE-assisted XRF method used in this study is also a novel, rapid, selective approach for heavy metal quantitation in complex matrices. Although specifically developed for this analysis, the method could be used in various applications to measure complexed and free metal species in the presence of each other.

## Conclusion

In conclusion, we showed that iron and copper can increase the yield of non-enzymatic glycation in an aqueous system. Reaction parameters, such as pH, reaction time, and temperature also affected the yield of the reaction. Methods were developed to quantify glycation products in simple and complex mixtures. A French fry model was used to assess the impact of iron on non-enzymatic glycation. Adding rice bran, rich in phytic acid and other NCAs, to potato decreased the amount of iron and glycation products in French fries. Phytic acid alone did not have the same effect on glycation, even though it decreased iron. An early glycation product, fructosyl-phenylalanine, was identified in all French fry samples. It was least abundant in the fries to which phytic acid was added prior to cooking. While more studies are required to confirm the promoting effect of iron on non-enzymatic glycation, our data suggest that a very strong positive correlation exists between iron and total glycation product concentration. Our ongoing study on metal-induced glycation in human cells could provide more insight into the potential impact of metal chelation on non-enzymatic glycation and its deleterious consequences to humans.

## Supplementary Material

mfae059_Supplemental_File

## Data Availability

The data underlying this article are available in the EMBL-EBI MetaboLights database [44] at https://www.ebi.ac.uk/metabolights/ and can be accessed with the identifier MTBLS10821.

## Notes

The authors dedicate this article to Professor Vladimir Pitchko (emeritus) for his tireless support of undergraduate chemistry research at Concordia University of Edmonton.
