# Supplementary material for: Assessing metal-induced glycation in French fries
Source: Metallomics. 2024 Dec 30;17(1):mfae059. doi: 10.1093/mtomcs/mfae059 (PMC11704954; doi:10.1093/mtomcs/mfae059)
Supplement: mfae059_Supplemental_File [file mfae059_supplemental_file.pdf]

Assessing metal-induced glycation in French fries

Seth Nobert, Haley Wolgien-Lowe, Tamara Davis, Emma Paterson, Thérèse Wilson-Rawlins, and Makan Golizeh*

[1] *Department of Environmental and Physical Sciences, Faculty of Science,* [2] *Metals in Environment and Health (MEH) Research Cluster, Concordia University of Edmonton, Edmonton, Alberta, Canada*

* Corresponding author. Mailing address: 7128 Ada Boulevard NW, Edmonton, Alberta T5B 4E4 Canada.

E-mail address: makan.golizeh@concordia.ab.ca.

SUPPLEMENTARY DATA

FIGURE S1

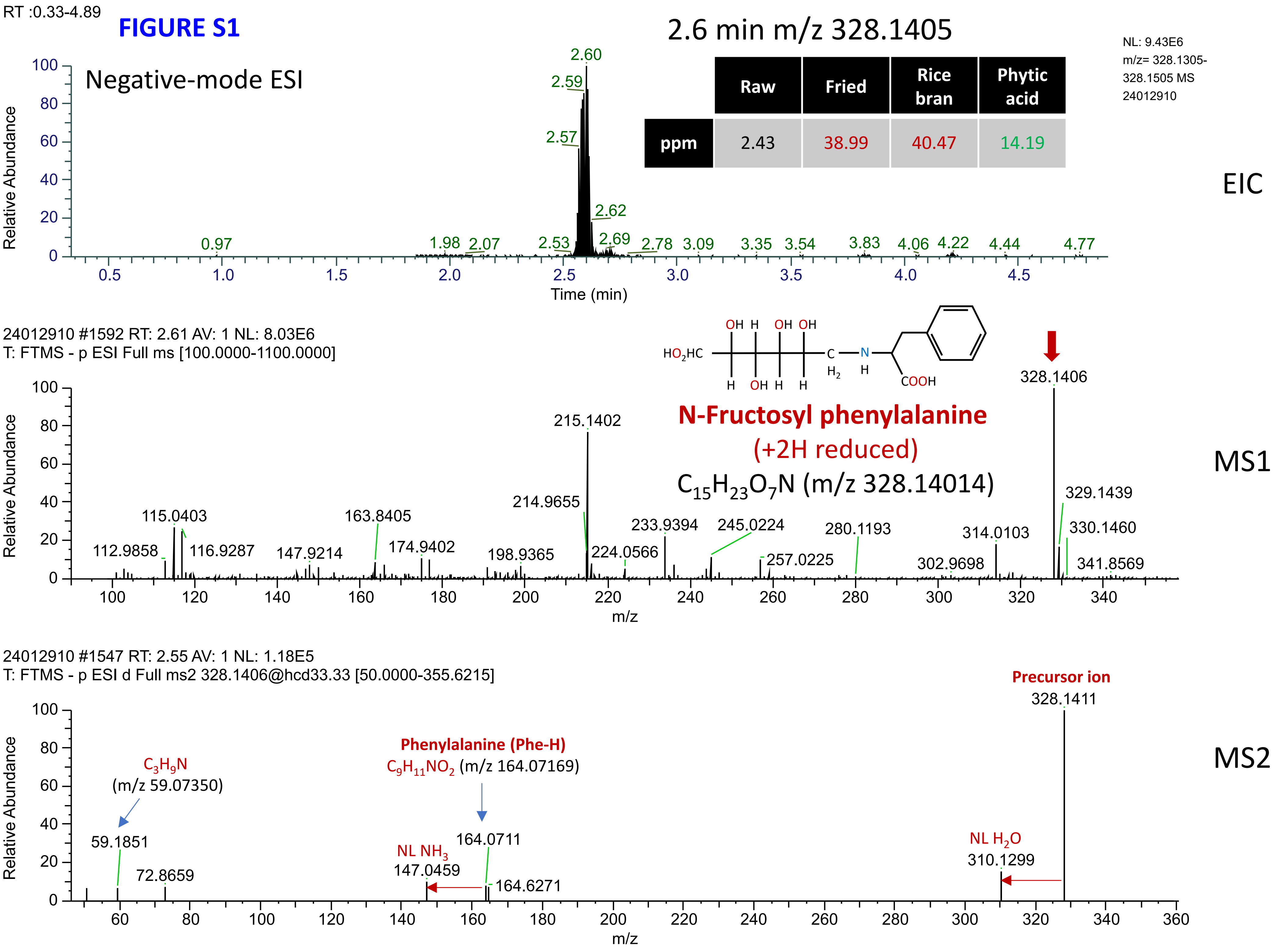

	Raw	Fried	Rice bran	Phytic acid
ppm	2.43	38.99	40.47	14.19

FIGURE S2

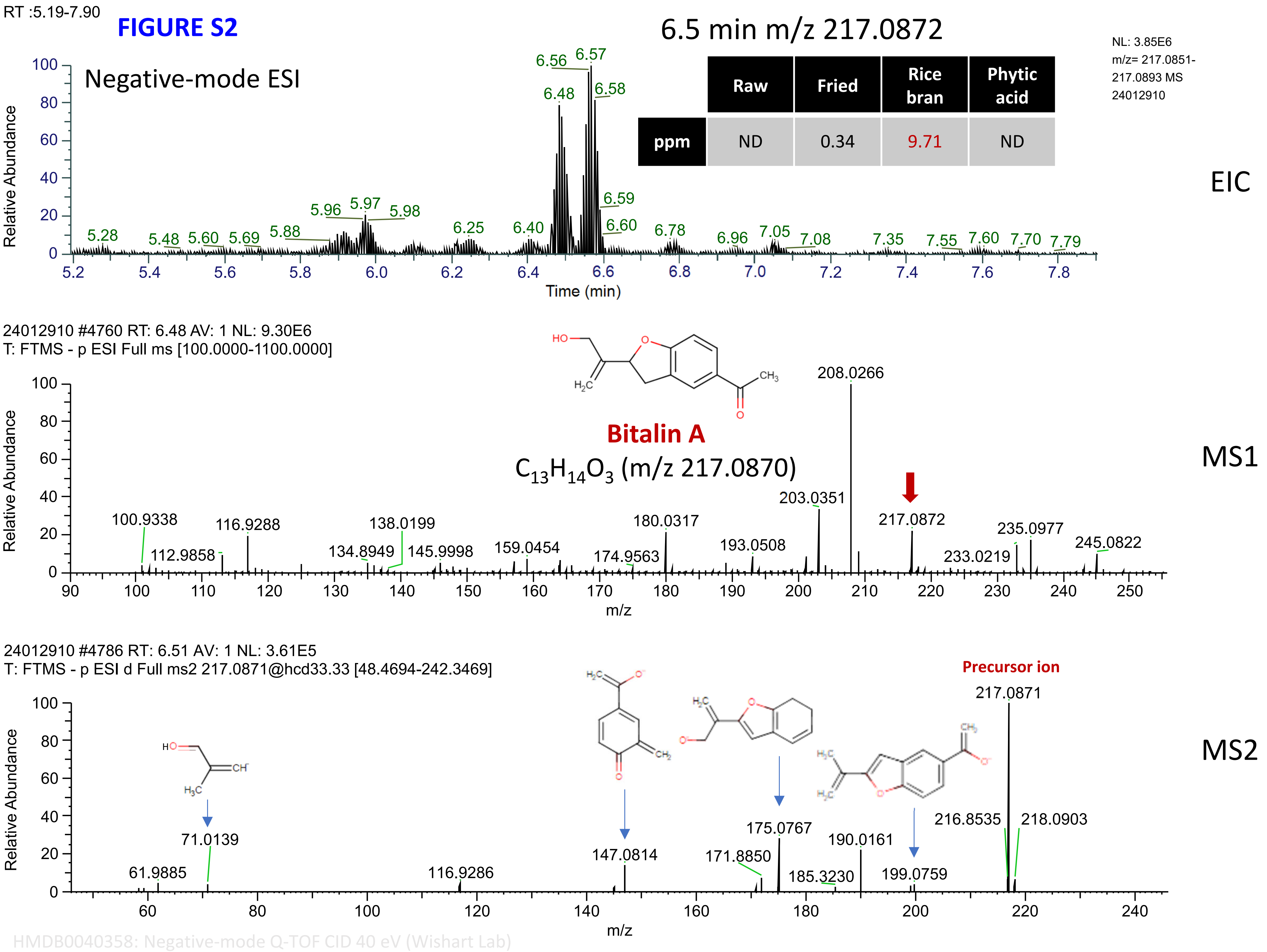

	Raw	Fried	Rice bran	Phytic acid
ppm	ND	0.34	9.71	ND

HMDB0040358: Negative-mode Q-TOF CID 40 eV (Wishart Lab)

FIGURE S3

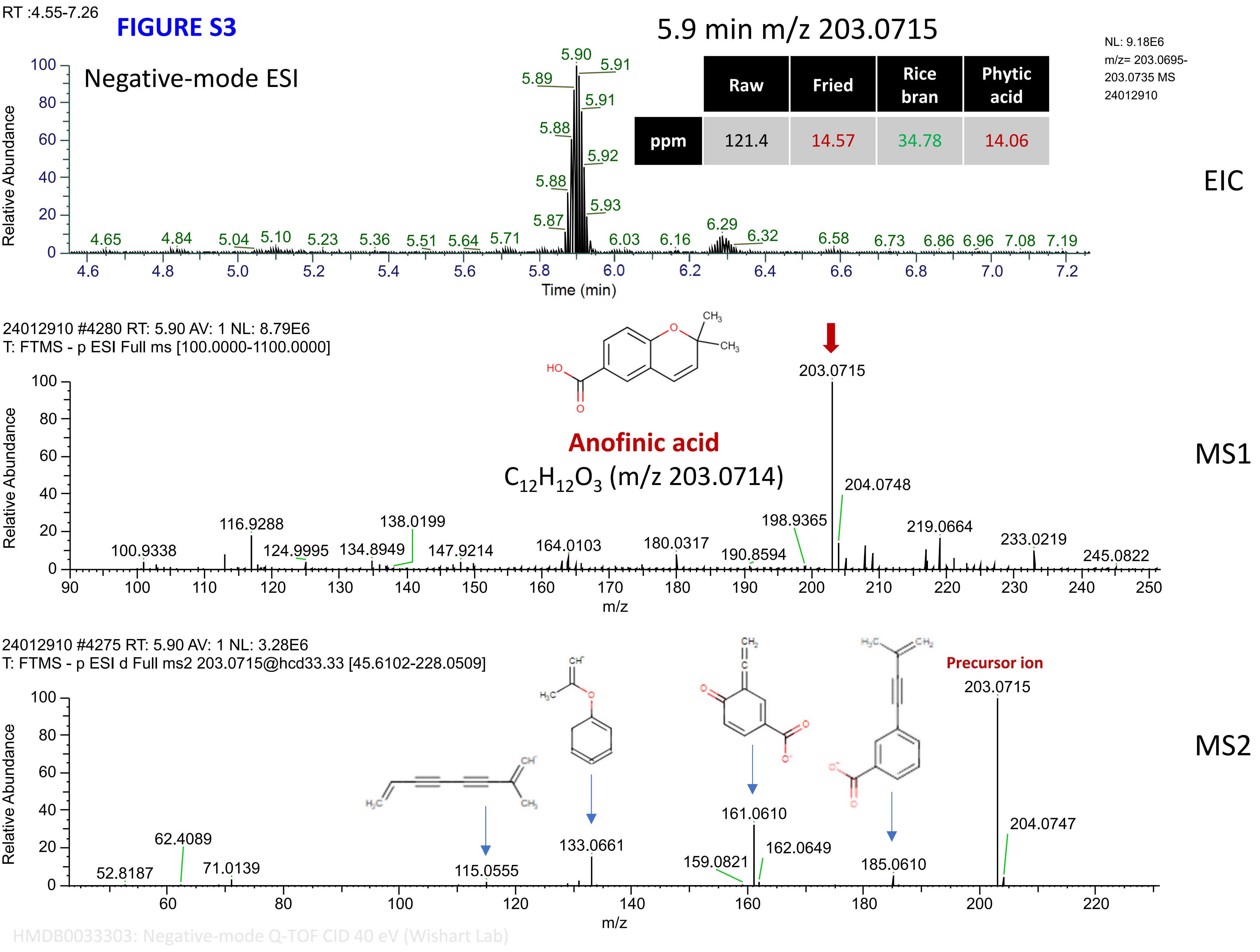
RT :4.55-7.26
5.9 min m/z 203.0715
NL: 9.18E6
m/z= 203.0695-203.0735 MS
24012910
Negative-mode ESI
Raw | Fried | Rice bran | Phytic acid
ppm | 121.4 | 14.57 | 34.78 | 14.06
EIC
Relative Abundance
Time (min)
24012910 #4280 RT: 5.90 AV: 1 NL: 8.79E6
T: FTMS - p ESI Full ms [100.0000-1100.0000]
Anofinic acid
$C_{12}H_{12}O_3$ (m/z 203.0714)
203.0715
204.0748
MS1
m/z
24012910 #4275 RT: 5.90 AV: 1 NL: 3.28E6
T: FTMS - p ESI d Full ms2 203.0715@hcd33.33 [45.6102-228.0509]
Precursor ion
203.0715
204.0747
161.0610
162.0649
159.0821
185.0610
133.0661
115.0555
71.0139
62.4089
52.8187
MS2
HMDB0033303: Negative-mode Q-TOF CID 40 eV (Wishart Lab)
